# “Near-TME”: proposed standardisation of the technique for proctectomy in male patients with ulcerative colitis

**DOI:** 10.1007/s10151-022-02579-z

**Published:** 2022-02-01

**Authors:** A. Garcia-Granero, G. Pellino, D. Fletcher-Sanfeliu, M. Millan, V. Primo-Romaguera, M. Garcia-Gausí, M. Fernandez, X. Gonzalez-Argente, A. Spinelli, A. Valverde-Navarro, E. Garcia-Granero

**Affiliations:** 1 Colorectal Unit, Hospital Universitario Son Espases, Palma de Mallorca, Spain; 2 Applied Surgical Anatomy Unit, Human Embryology and Anatomy Department, University of Valencia, Valencia, Spain; 3 Human Embryology and Anatomy Department, University of Islas Baleares, Palma, Spain; 4 Department of Advanced Medical and Surgical Sciences, Università Degli Studi Della Campania “Luigi Vanvitelli”, Naples, Italy; 5 Colorectal Surgery, Vall d’Hebron University Hospital, Barcelona, Spain; 6 Cardiac Surgery Department, Hospital Universitario Son Espases, Palma de Mallorca, Spain; 7 Colorectal Surgery, Hospital Universitario Y Politecnico “La Fe”, Valencia, Spain; 8 Department of Biomedical Sciences, Humanitas University, Pieve Emanuele, Milan, Italy; 9 IRCCS Humanitas Research Hospital, Rozzano, Milan Italy

**Keywords:** Ulcerative colitis, Proctectomy, Close rectal dissection, Intra-mesorectal, Inflammatory bowel diseases, Surgical anatomy

## Abstract

**Background:**

The aim of the present study was to describe in detail an approach to proctectomy in ulcerative colitis (UC), which can be standardized; near-total mesorectal excision (near-TME), to prevent injuries to autonomic pelvic nerves and subsequent sexual dysfunction.

**Methods:**

We demonstrate the technique ex vivo on a cadaver from a male patient in lithotomy position and on a sagittal section of a male pelvis. We also demonstrate the technique in vivo in two male patients diagnosed with UC, with no history of sexual dysfunction or bowel neoplasia. The study was performed at the Human Embryology and Anatomy Department. University of Valencia, Spain.

**Results:**

The posterolateral dissection during a near-TME is similar to that of an oncologic TME, whereas the anterolateral is similar to that of a close rectal dissection. The near-TME technique preserves the superior hypogastric plexus, the hypogastric nerves, the *nervi erigentes*, the inferior hypogastric plexus, the pelvic plexus and the cavernous nerves.

**Conclusion:**

The near-TME technique is the standardisation of the technique for proctectomy in male patients with ulcerative colitis. Near-TME requires experience in pelvic surgery and an exhaustive knowledge of the embryological development and of the surgical anatomy of the pelvis.

**Supplementary Information:**

The online version contains supplementary material available at 10.1007/s10151-022-02579-z.

## Introduction

Among the proposed techniques for proctectomy in patients with ulcerative colitis (UC), the most frequently described is total mesorectal excision (TME) [1], commonly used during oncologic rectal resection and close rectal dissection (CRD) [2].

Authors of a recent position statement of the Italian Society of Colorectal Surgery (SICCR) advised that a plane of dissection close to the rectum or an incomplete mesorectal excision is preferred to preserve fertility and avoid nerve injuries, if no rectal cancer or dysplasia of any degree is present in the rectum [3]. Some authors confute such an approach, for different reasons, including the risk of rectal injury, and intraoperative bleeding that makes dissection difficult [4]. The European Crohn’s and Colitis Organisation (ECCO) consensus on surgery for UC, recommended anterolateral close rectal dissection, in combination with a posterior dissection in the TME plane [5]. The definition of this technique has not been standardised, meaning that a variety of definitions are used (e.g. “badTME”, CRD, intramesorectal dissection, incomplete mesorectal excision), even if only a few of them have been clearly described, suggesting that its surgical steps might need to be clarified.

The aim of the present study was to describe in detail an approach to proctectomy in UC, which can be standardised, the near-total mesorectal excision (near-TME), to prevent injuries to autonomic pelvic nerves and subsequent sexual dysfunction.

## Surgical technique

In video 1, we demonstrate the technique on a cadaver from a male patient in lithotomy position and on a sagittal section of a male pelvis. In video 2, we demonstrate near-TME in two male patients diagnosed with UC, with no history of bowel neoplasia.

**Fig. 1:**
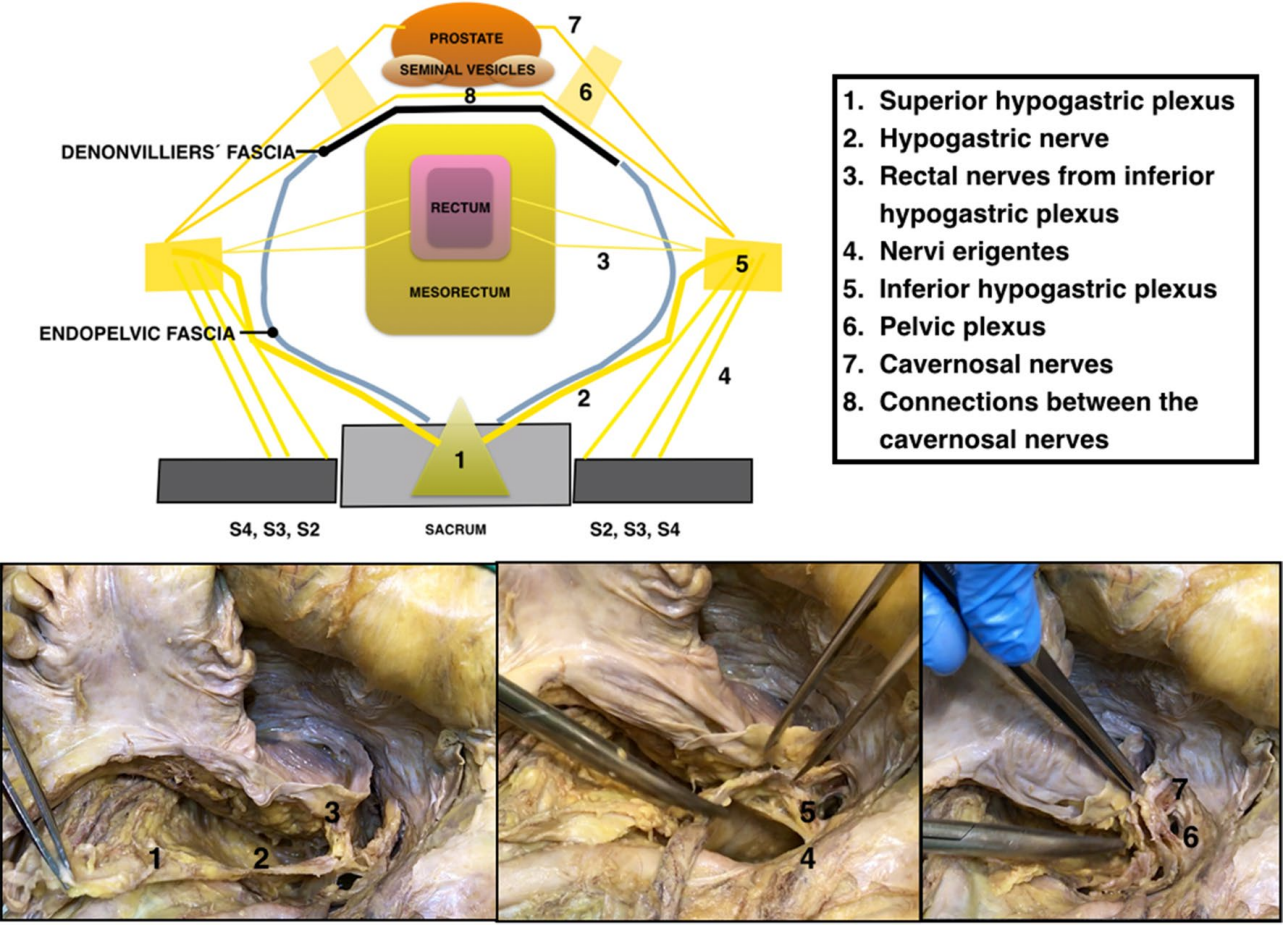
Drawing and cadaveric dissection showing male pelvic autonomic nerves. 1: Superior hypogastric plexus, 2: Hypogastric nerves, 3: Rectal nerves from inferior hypogastric plexus, 5: Inferior hypogastric plexus, 6: Pelvic plexus, 7: Cavernous nerves, 8: Connections between the cavernous nerves

The injury of the autonomic nerves during TME can occur at five sites: the superior hypogastric plexus and the hypogastric nerves can be damaged during the posterior dissection of the mesorectum, at the level of the promontory; the *nervi erigentes* can be injured during posterior dissection, at the level of S3-S4; the inferior hypogastric plexus is threatened during the lateral mesorectal dissection, the cavernous nerves during anterolateral dissection, and the connections of cavernous nerves of both sides during the anterior dissection [6] (Fig. 1).

**Fig. 2:**
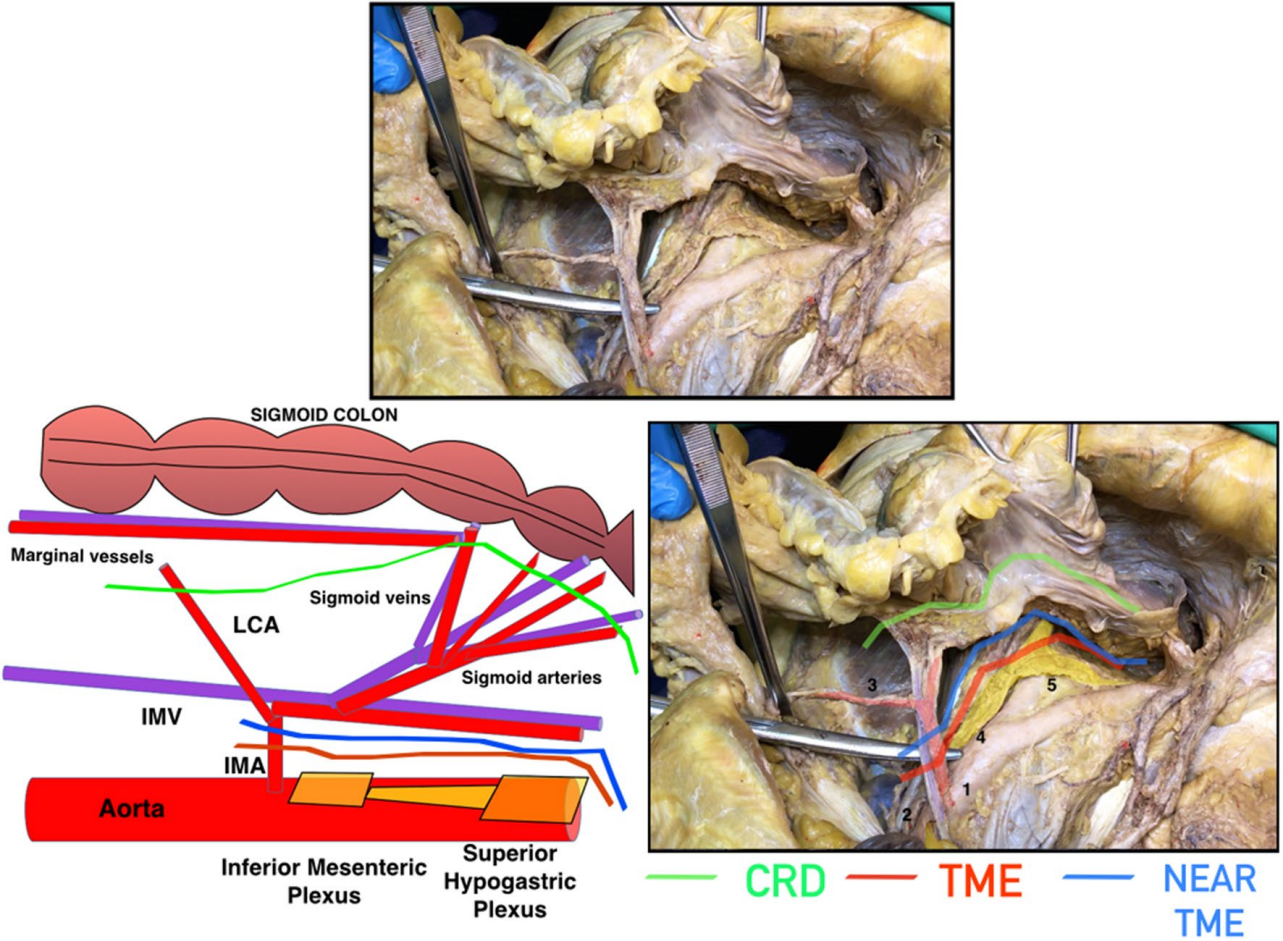
Drawing and cadaveric dissection comparing close rectal dissection (CRD), total mesorectal excision (TME) and near-TME during inferior mesenteric plexus and superior hypogastric plexus preservation. 1: Inferior mesenteric artery (IMA), 2: Inferior mesenteric vein (IMV), 3: Left colic artery (LCA), 4: Inferior mesenteric plexus, 5: Superior hypogastric plexus

**Fig. 3:**
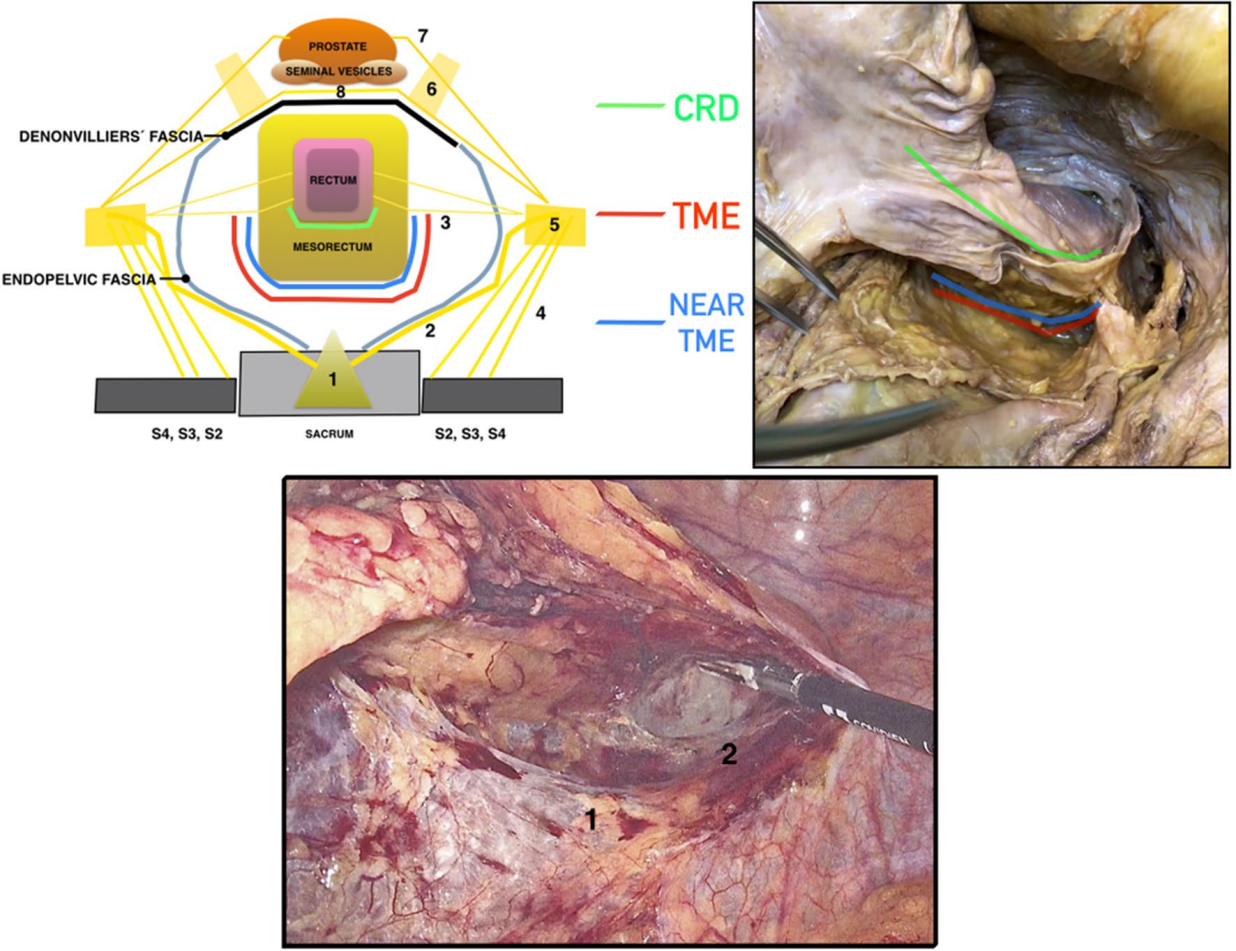
Drawing, cadaveric, and laparoscopic dissection comparing close rectal dissection (CRD), total mesorectal excision (TME) and near-TME during posterior pelvic rectal dissection. 1: Superior hypogastric plexus, 2: Hypogastric nerves, 3: Rectal nerves from the inferior hypogastric plexus, 5: Inferior hypogastric plexus, 6: Pelvic plexus, 7: Cavernous nerves, 8: Connections between the cavernous nerves

To preserve the superior hypogastric plexus, during CRD, the dissection starts close to the wall of the sigmoid colon, and ligation of the terminal branches of the sigmoid vessels is carried out [1]. The dissection during near-TME is started below the superior rectal artery, and the plexus must be identified at the level of the mesorectum (Fig. 2). The ureterohypogastric fascia/endopelvic fascia is a useful landmark to preserve both hypogastric nerves [7]. Since this fascia contains the hypogastric nerves, performing the dissection between the mesorectal fascia and the ureterohypogastric/endopelvic fascia ensures that injuries to hypogastric nerves are avoided. Posterior dissection is performed along this plane, continued downward to reach the *levator ani*, as no other nerve structures are encountered following this route (Fig. 3).

The *nervi erigentes* runs laterally to the ureterohypogastric fascia, and then joins the hypogastric nerves to form the hypogastric pelvic plexus. Such nerve connections are lateral to the ureterohypogastric fascia, so that they can be preserved if the posterolateral dissection is continued between the mesorectal and the ureterohypogastric fascia.

**Fig. 4:**
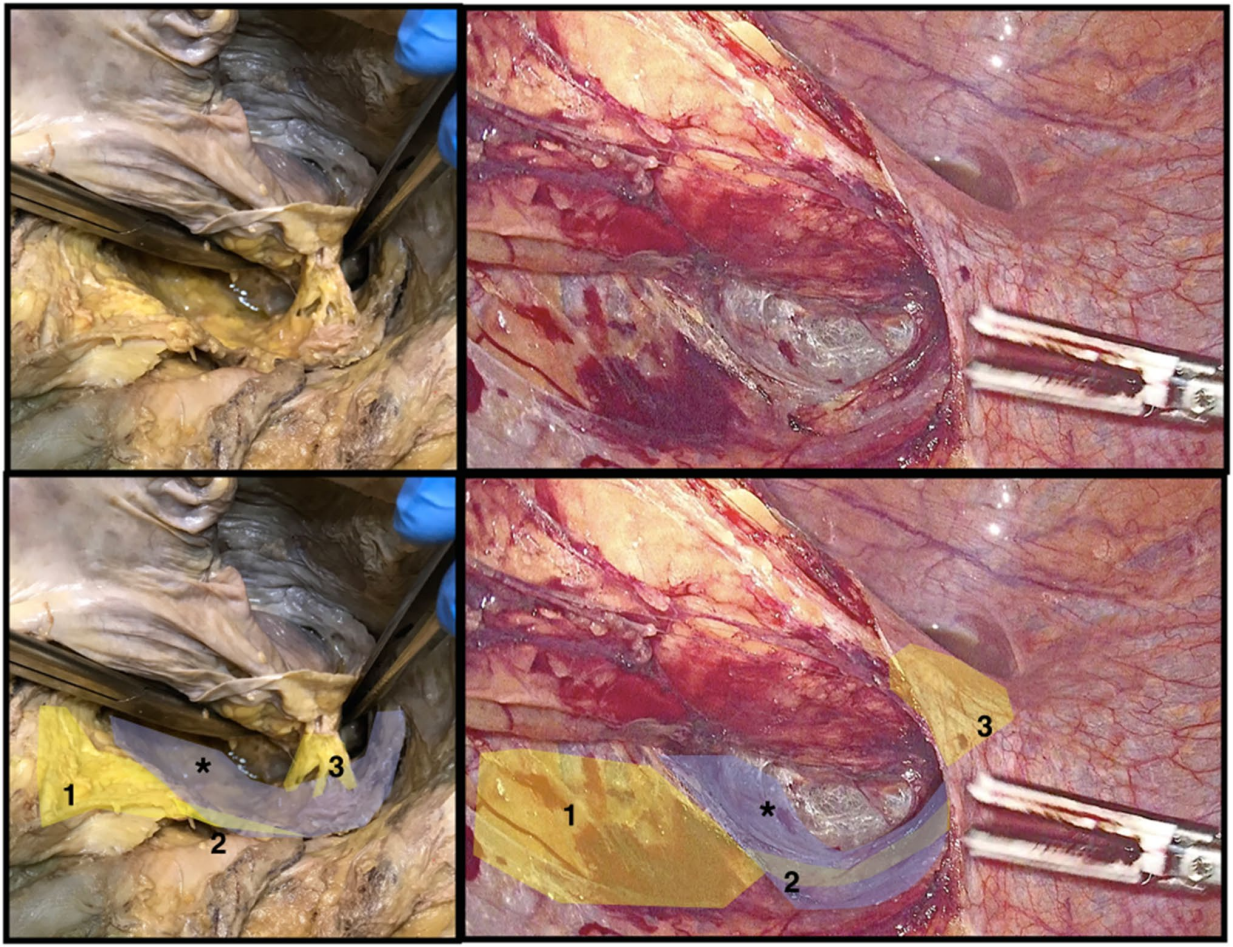
Cadaveric and laparoscopic dissection showing the area of the inferior rectal nerves. Nerves coming from the inferior hypogastric plexus to the posterolateral wall of the rectum. 1: Superior hypogastric plexus, 2: Hypogastric nerves, 3: Rectal nerves from inferior hypogastric plexus, *endopelvic/ureterohypogastric fascia

The posterolateral dissection between the mesorectum and the ureterohypogastric fascia, continues until inferior rectal nerves area (Fig. 4). The inferior rectal nerves come from inferior hypogastric plexus and run to the posterolateral wall of the rectum, most often, accompanied by the middle rectal artery.

**Fig. 5:**
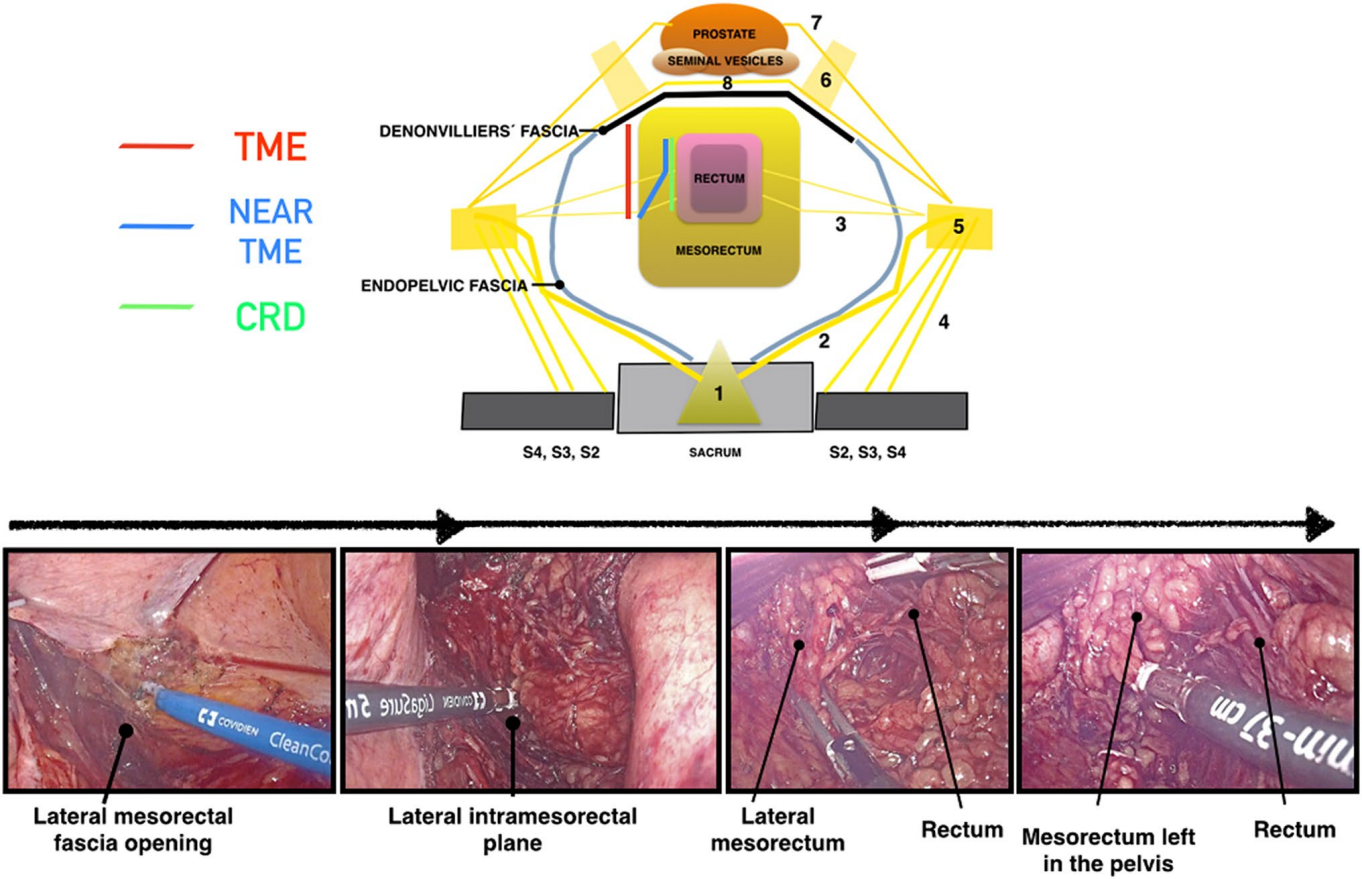
Drawing and laparoscopic dissection comparing close rectal dissection (CRD), total mesorectal excision (TME) and near-TME during lateral pelvic rectal dissection. 1: Superior hypogastric plexus, 2: Hypogastric nerves, 3: Rectal nerves from inferior hypogastric plexus, 5: Inferior hypogastric plexus, 6: Pelvic plexus, 7: Cavernous nerves, 8: Connections between the cavernous nerves

At this level, the lateral mesenteric fascia is opened, to achieve access to the intra-mesorectal plane, avoiding injuries to the inferior hypogastric plexus (Fig. 5).

**Fig. 6:**
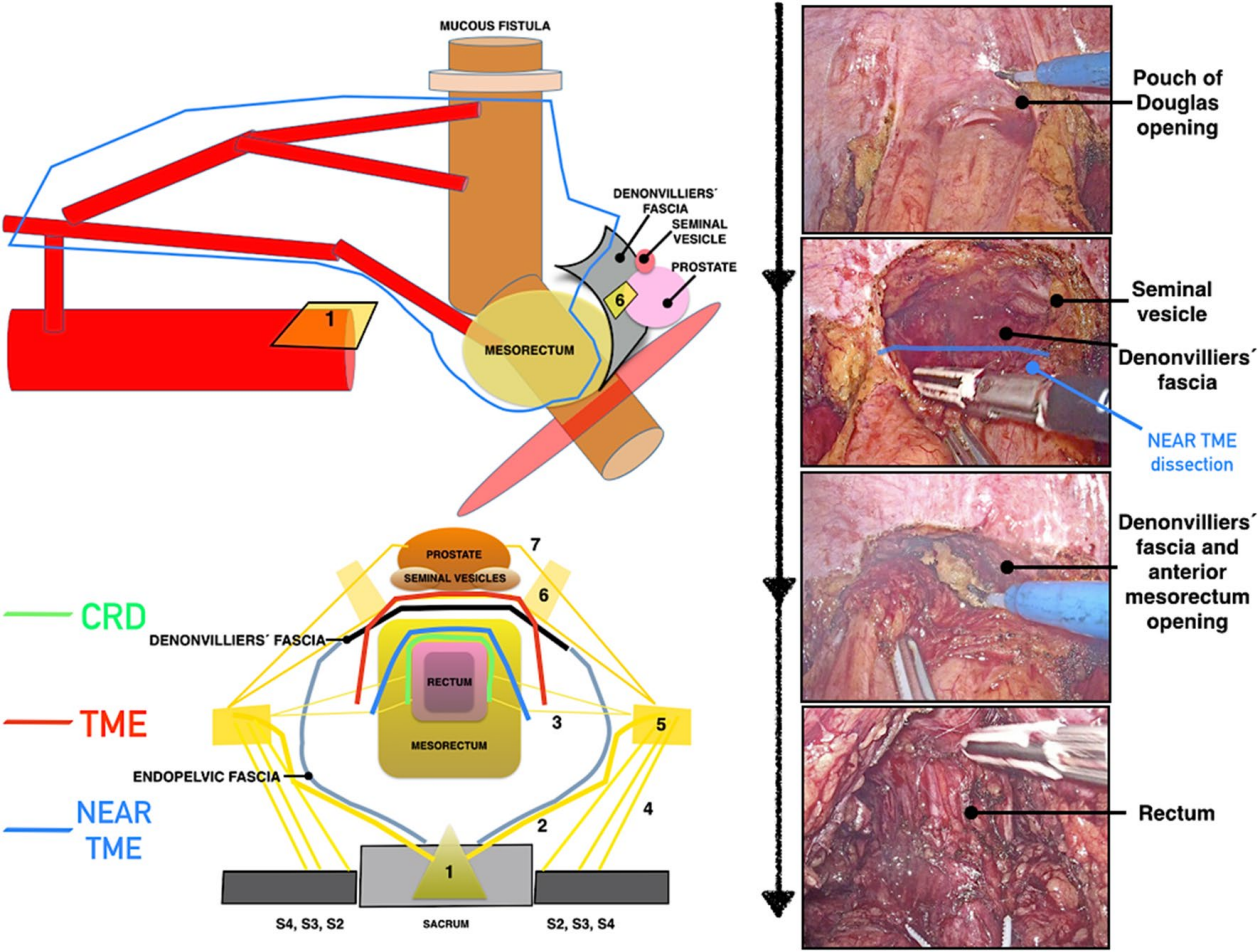
Drawing and laparoscopic dissection comparing close rectal dissection (CRD), total mesorectal excision (TME) and near-TME during anterior pelvic rectal dissection. 1: Superior hypogastric plexus, 2: Hypogastric nerves, 3: Rectal nerves from inferior hypogastric plexus, 5: Inferior hypogastric plexus, 6: Pelvic plexus, 7: Cavernous nerves, 8: Connections between the cavernous nerves

Denonvilliers’ fascia can be used as a landmark to avoid injuries to the cavernosal nerves and their connections. This fascia runs from the pouch of Douglas down to the *levator ani*. The rectum lies posterior to Denonvilliers’ fascia, and the seminal vesicles and the prostate are anterior to the fascia. The cavernosal nerves originate from the inferior hypogastric plexus and run anterolaterally to Denonvilliers’ fascia. The connections between the cavernous nerves of both sides are also anterior to the fascia [8]. During anterior pelvic dissection, the structure should be identified and dissected for no more than 1 cm, to open the pouch of Douglas**.** The cavernous nerves and their connections are preserved if the anterolateral dissection is performed by entering the mesorectum, following the limited opening in Denonvilliers’ fascia (Fig. 6).

**Fig. 7:**
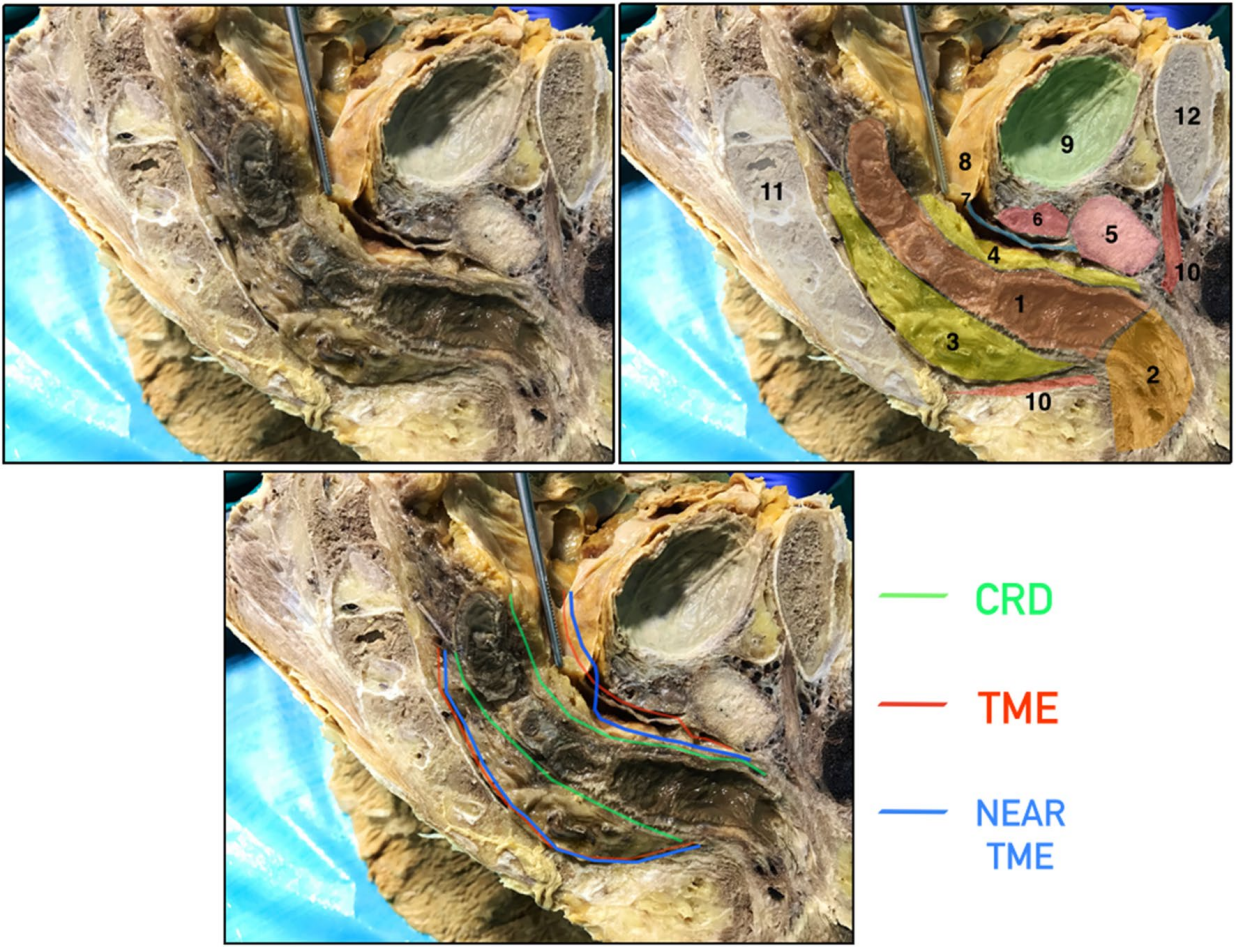
Male sagittal pelvis. Drawing and cadaveric dissection comparing close rectal dissection (CRD), total mesorectal excision (TME) and near-TME. 1: Rectum, 2: Anal canal, 3: Posterior mesorectum, 4: Anterior mesorectum, 5: Prostate, 6: Seminal vesicle, 7: Denonvilliers´ fascia, 8: Pouch of Douglas, 9: Bladder, 10: Levator ani muscle, 11: Sacrum, 12: Pubis

Lastly, the remaining anterior dissection is performed following the perirectal plane (Fig. 7), whereas Heald et al. in their original description of the technique considered that Denonvilliers fascia should be resected in TME surgery [1].

**Fig. 8:**
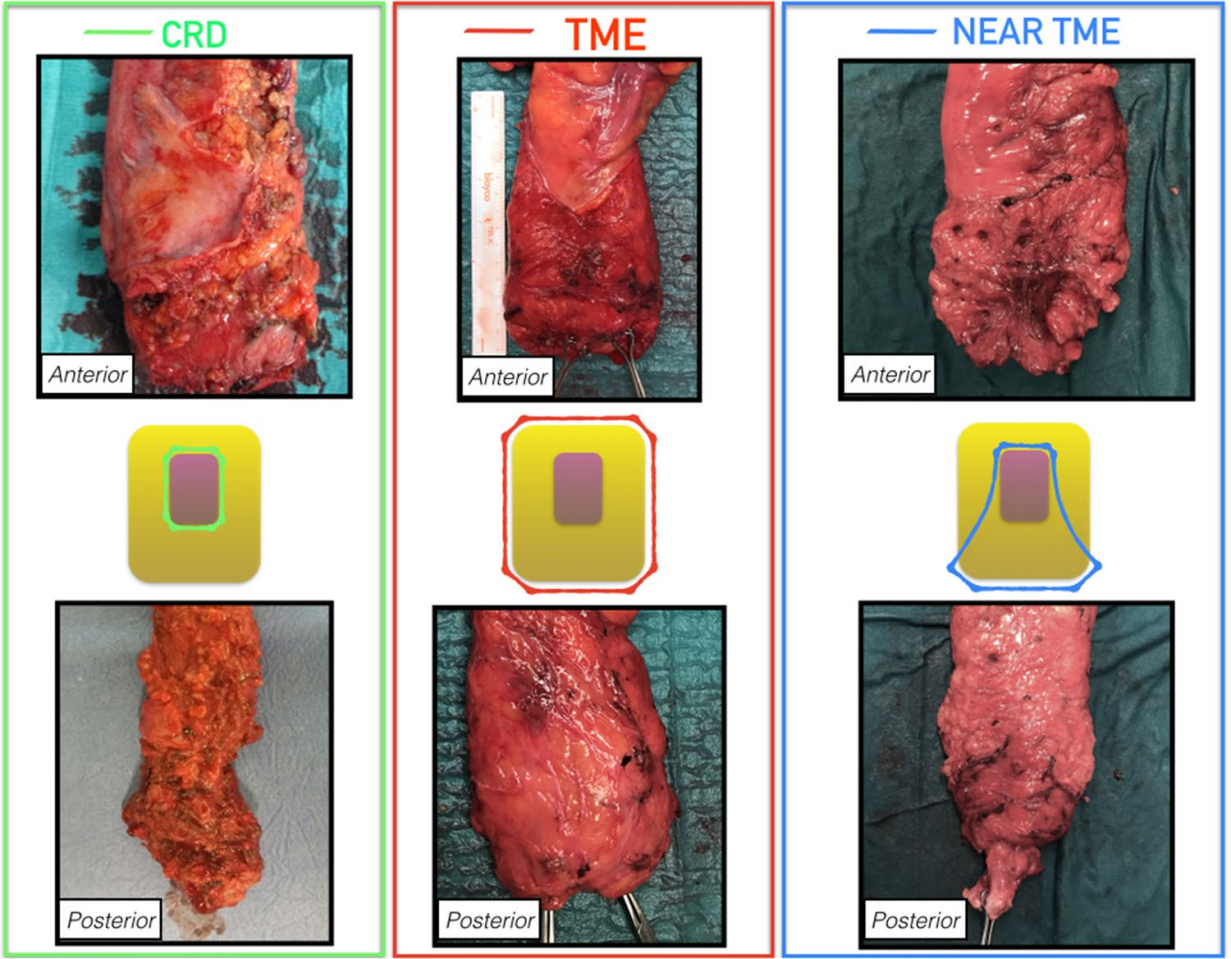
Surgical specimen of proctectomy comparing close rectal dissection (CRD), total mesorectal excision (TME) and near-TME. (CRD specimen: courtesy of Dr Antonino Spinelli, Humanitas Research Hospital, Rozzano (MI), Italy; TME specimen from a rectal cancer procedure, courtesy of Dr Eduardo Garcia-Granero, Hospital Unviersitario y Politecnico La Fe, Valencia, Spain)

At this stage, the entire rectal circumference has been dissected, and the rectum can be divided (Fig. 8).

## Discussion

In male patients, retrograde ejaculation and erectile dysfunction are the main sexuality- and fertility-related complications that may occur after an ileal pouch–anal anastomosis [5].

TME is associated with higher risk of injury to autonomic pelvic nerves [6]. CRD consists of a perirectal dissection, with the aim of reducing the risk of injury to these nerves [2]. Furthermore, some authors argue that the retained mesorectal fat would allow for better pelvic filling and could reduce the risk of a pre-sacral sinus if the anastomosis leaks [9]. Other authors confute such an approach, for different reasons, including the risk of rectal injury, intraoperative bleeding that makes dissection difficult, and the consequences of leaving an inflamed or affected mesorectum in the pelvis of patients with inflammatory bowel disease since, in the opinion of some authors, this might impact negatively pouch function [4]. Even if the latter might be more relevant in patients with Crohn’s disease than in those with UC, it should be considered that a proportion of patients might be diagnosed with Crohn’s disease after pouch construction [10].

The near-TME dissection relies on a perfect knowledge of the surgical anatomy of the rectum, mesorectum, autonomic pelvic nerves, and their relationship with pelvic structures. In order to safely implement such standardised procedure, this knowledge can be obtained by surgeons during surgical simulation on cadavers. Then, surgeons learning near-TME should be supervised by surgeons with experience in minimally invasive and inflammatory bowel disease surgery.

The posterolateral dissection between the mesorectum and the ureterohypogastric fascia, makes it possible to protect the superior hypogastric plexus, the hypogastric nerves and the inferior hypogastric plexus, and the *nervi erigentes*. To avoid injuries to the pelvic plexus, the cavernous nerves and their connection with the contralateral bundle, the dissection must be performed posterior to Denonvilliers’ fascia. However, the anterior mesorectum is much thinner and less represented. Hence, during near-TME, the rectal serosa should be identified, to avoid damaging the above reported nerve structures.

## Conclusion

Near-TME is a proposed standardisation of the technique for proctectomy in male patients with ulcerative colitis, which can potentially reduce the risks of nerve injuries and bleeding, provided that this is performed by adequately trained surgeons. The posterolateral dissection is similar to that of an oncologic TME, whereas the anterolateral is similar to that of a CRD.

## Supplementary Information

Below is the link to the electronic supplementary material.

Supplementary file1 (MP4 153748 kb)

Supplementary file2 (MP4 416217 kb)

Supplementary file3 (DOCX 11 kb)
