# Supplementary material for: “Near-TME”: proposed standardisation of the technique for proctectomy in male patients with ulcerative colitis
Source: Tech Coloproctol. 2022 Feb 1;26(3):217–26. doi: 10.1007/s10151-022-02579-z (PMC8857132; doi:10.1007/s10151-022-02579-z)
Supplement: Supplementary file 3 — Supplementary file3 (DOCX 11 kb) [file 10151_2022_2579_MOESM3_ESM.docx]

**Supplementary Material**

**Video 1.** Cadaveric dissection with demonstration of the excision planes available for proctectomy, with focus on near-total mesorectal excision (near-TME) plane.

**Video 2.** Laparoscopic completion proctectomy, performed using the near-total mesorectal excision (near-TME) technique.
